# Reply to Pugnaloni et al. Comment on “Othman et al. Ventricular Topology in Congenital Heart Defects Associated with Heterotaxy: Can We Find Patterns Reflecting the Syndrome-Specific Tendency for Visceral Symmetry? *J. Cardiovasc. Dev. Dis.* 2025, *12*, 430”

**DOI:** 10.3390/jcdd13020068

**Published:** 2026-01-27

**Authors:** Jörg Männer, Talat Mesud Yelbuz

**Affiliations:** 1 Institute for Anatomy and Cell Biology, UMG, Georg-August-University of Göttingen, D-37075 Göt-tingen, Germany; jmaenne@gwdg.de; 2 King Abdulaziz Cardiac Center, Ministry of National Guard Health Affairs, Riyadh 11426, Saudi Arabia; 3 King Abdullah International Medical Research Center (KAIMRC), Riyadh 11426, Saudi Arabia; 4 Department of Cardiac Sciences, King Saud Bin Abdulaziz University for Health Sciences, Riyadh 11426, Saudi Arabia

We greatly appreciate the thoughtful and detailed comments provided by Bruno Marino and his colleagues [1] regarding our recent study on ventricular topology in congenital heart defects associated with heterotaxy [2]. Their emphasis on the importance of precise anatomical description, particularly of the infundibular morphology, highlights a critical dimension in the ongoing effort to refine our pathogenetic understanding of these complex malformations.

In our above-mentioned study, we used the diagnostic term double outlet right ventricle (DORV) to characterize a group of congenital heart defects (CHDs) in which both arterial trunks arise from the morphologically right ventricle. Our usage of this diagnostic term complies with its current usage in daily medical practice and in the vast majority of previous studies on congenital heart defects (CHDs) associated with heterotaxy. Marino and coauthors have made the comment that, in their opinion, the use of the term DORV to describe the cardiac outflow tract of heterotaxy patients is “… insufficient and potentially misleading. DORV, accordingly with the sequential segmental analysis, defines a ventriculo-arterial connection but, by itself, does not represent a precise anatomic description of the cardiac outflow tracts” [1].

We fully agree that the diagnostic term DORV, as applied in ours as well as in the vast majority of previous studies on CHDs associated with heterotaxy, does not characterize a morphologically homogeneous group of congenital malformations. Since the introduction of DORV as a diagnostic term by Witham in 1957 [3], those dealing with the morphology of CHDs have learnt that this term does not define a specific morphological entity, but simply represents the ventriculo-arterial connections in which both arterial trunks arise from the morphologically right ventricle (for a review see [4]). The two senior authors of our study (J.M. and T.M.Y.) have emphasized this fact in a recent book chapter dealing with pathogenetic key concepts of CHDs [5]. In this book chapter—integrated right at the start of the new Manual of Pediatric Cardiac Care (with T.M.Y. as Editor-in-Chief), published in 2024 in two volumes by Springer-Nature—it is furthermore emphasized that the diagnosis DORV encompasses a continuum spectrum of congenital heart defects, which includes three embryologically interesting pathomorphological phenotypes:

- Phenotype 1: DORV with abnormally related great arteries arranged in a parallel (’non-spiralized’) fashion (with an anterior aorta associate with a subaortic infundibulum);
- Phenotype 2: DORV with abnormally related great arteries arranged in a side-by-side fashion (with a right- or left-sided aorta associate with bilateral infundibulums);
- Phenotype 3: DORV with normally related (’spiralized’) great arteries (with a posterior aorta associate with a subpulmonary infundibulum).

Each of these phenotypes might represent an example for a certain pathogenetic track leading to the ventriculo-arterial connections of DORV. The abnormal positional relationships of the great arteries and abnormal infundibular morphologies characterizing the first two phenotypes of DORV strongly suggest that impaired development of the outflow tract of the embryonic heart (formerly called the conotruncus) plays a fundamental role in the pathogenesis of these malformations. This idea is supported by data from several animal models with disturbed development of the embryonic cardiac outflow tract (e.g., cranial neural crest-ablation models). It is therefore no wonder that CHDs with DORV usually are assigned to the pathogenetic group of so-called conotruncal heart defects, which also includes TGA, congenitally corrected TGA, and ToF.

The normal positional relation of the great arteries and normal infundibular morphology found in the third phenotype of DORV, however, does not seem to justify the assignment of such CHDs to the pathogenetic category of conotruncal heart defects [5,6]. Data from human pathological heart collections as well as embryological data suggest that abnormal development of the ventricular inflow of the embryonic heart (AV-canal) rather than ventricular outflow seem to play a fundamental role in the pathogenesis of this DORV phenotype [7,8,9,10]. These findings not only underscore the need for accurate description of the anatomy of CHDs with DORV but also for caution when assigning such malformations reflexively to the pathogenetic group of conotruncal defects, as such classification may obscure the true developmental mechanisms involved [5,6].

Complex CHDs occurring in the setting of heterotaxy syndrome usually are assigned to the pathogenetic group of CHDs resulting from abnormal left–right patterning of the lateral plate mesoderm and heart looping [5,11]. Heart looping is a complex morphogenetic process that transforms the initially straight embryonic heart tube into a looped heart tube of complex helical (chiral) configuration [12,13]. This process not only determines the definitive topology (chirality) of the ventricular heart segment (ventricular right-hand topology or ventricular left-hand topology) but also seems to contribute to the topogenesis (torsion) of the ventricular outflow and great arterial trunks [14]. Thereby, the sidedness of ventricular topology seems to be determined by the emergence of subtle morphological left–right asymmetries at the arterial and venous poles of the pre-looping embryonic heart [15,16], whereas the spiraling of the arterial trunks seems to arise mainly from the growth behavior of the embryonic outflow tract [14,17]. Thus, the topogenesis of the ventricular heart segment and the helical morphogenesis of the ventricular outflow and arteries are two different components of the looping process. In some aspects they are linked with each other while in other aspects they are not linked with each other. If, for example, the arterial segment shows a helical configuration (spiraling), its sense of winding is typically linked to ventricular topology. This means that the normal right-handed spiraling of the great arteries is typically linked with ventricular right-hand topology, as found in situs solitus hearts, whereas an abnormal left-handed spiraling of the great arteries is typically linked with ventricular left-hand topology, as found in situs inversus hearts. On the other hand, absence of the spiraling of the great arteries, which is typically found in cases of TGA or DORV with parallel great arteries, is not linked with absence of a handed (chiral) arrangement of the two ventricular heart chambers. This means that neither the presence nor the type of chiral arrangement of the ventricular heart chambers (right-hand or left-hand topology) can be made responsible for the presence or absence of a spiral (helical) arrangement of the great arteries. This is documented by epidemiological data from families with genetically inherited cases of TGA published by Bruno Marino’s group [18]. In such families, discordant ventriculo-arterial connections can occur in association with ventricular right-hand topology (TGA) as well as ventricular left-hand topology (congenitally corrected TGA).

The focus of our above-mentioned study was on ventricular topology. Its main goal was to collect and analyze data from human patients with CHDs that might help to identify some of the biomechanical factors determining the topology (chirality) of the ventricular heart segment, which normally is characterized by a strong bias towards the right-handed arrangement. For this purpose, we have determined the statistical distribution pattern of ventricular right-hand and left-hand topology in a relatively large group of human patients with CHDs in the setting of heterotaxy. The data were primarily used to check the validity of two embryological concepts currently used to explain the chiral morphogenesis of the ventricular heart segment. Our study was not designed to collect data that might refine our mechanistic understanding of the helical (chiral) morphogenesis of the arterial heart segment. As outlined above, ventricular topology is not linked to the presence or absence of spiralized great arteries. Therefore, for our above-mentioned retrospective study, we did not see a need for a detailed morphological characterization of those CHDs affecting the cardiac outflow tract (formerly called conotruncal malformations).

We appreciate Marino et al.’s call for more detailed morphological characterization of the cardiac outflow segment (infundibulum, great arterial trunks) in heterotaxy-associated CHDs. Their observations regarding differences between the two subsets of the syndrome—particularly the frequent association of RAI with TGA and DORV with parallel and non-spiralized great arteries, versus the more balanced ventriculo-arterial concordance in LAI—are highly valuable. These striking differences between the two subsets of the heterotaxy syndrome indeed might contribute not only to differences in surgical prognosis but also might contribute to refining our understanding of the etiology and morphogenesis of CHDs traditionally assigned to the pathogenetic group of conotruncal heart defects, and therefore should be integrated into future morphogenetic analyses.

In conclusion, we concur that some diagnostic terms currently used for the characterization of complex CHDs, such as DORV, do not provide a morphologically accurate diagnosis and therefore should be complemented by precise anatomical descriptions or classifications to advance our morphogenetic understanding of isolated as well as syndrome-associated CHDs. We see the comments made by Marino et al. as an important contribution that reinforces the necessity of integrating detailed morphological analysis with embryological and genetic perspectives. This approach may help refine both clinical classification and pathogenetic research in the field of CHDs.
